# IL-6 signaling blockade increases inflammation but does not affect muscle function in the *mdx* mouse

**DOI:** 10.1186/1471-2474-13-106

**Published:** 2012-06-20

**Authors:** Matthew C Kostek, Kanneboyina Nagaraju, Emidio Pistilli, Arpana Sali, San-Huei Lai, Brad Gordon, Yi-Wen Chen

**Affiliations:** 1 Laboratory of Muscle and Translational Therapeutics, Department of Exercise Science, University of South Carolina, Columbia, SC, USA; 2 Department of Integrative Systems Biology, George Washington University, Children’s National Medical Center, Washington, DC, USA; 3 University of Pennsylvania School of Medicine, Philadelphia, Pennsylvania, USA; 4 Laboratory of Muscle and Translational Therapeutics, 921 Assembly St. / PHRC / 3rd floor, Columbia, SC, USA; 5 111 Michigan Ave. NW, Department of Integrative Systems Biology, George Washington University School of Medicine, Washington, DC, USA

**Keywords:** IL-6, Muscular dystrophy, Inflammation, Duchenne

## Abstract

**Background:**

IL-6 is a pleiotropic cytokine that modulates inflammatory responses and plays critical roles in muscle maintenance and remodeling. In the mouse model (*mdx*) of Duchenne Muscular Dystrophy, IL-6 and muscle inflammation are elevated, which is believed to contribute to the chronic inflammation and failure of muscle regeneration in DMD. The purpose of the current study was to examine the effect of blocking IL-6 signaling on the muscle phenotype including muscle weakness and pathology in the *mdx* mouse.

**Methods:**

A monoclonal antibody against the IL-6 receptor (IL-6r mAb) that blocks local and systemic IL-6 signaling was administered to *mdx* and BL-10 mice for 5 weeks and muscle function, histology, and inflammation were examined.

**Results:**

IL-6r mAb treatment increased *mdx* muscle inflammation including total inflammation score and ICAM-1 positive lumens in muscles. There was no significant improvement in muscle strength nor muscle pathology due to IL-6r mAb treatment in *mdx* mice.

**Conclusions:**

These results showed that instead of reducing inflammation, IL-6 signaling blockade for 5 weeks caused an increase in muscle inflammation, with no significant change in indices related to muscle regeneration and muscle function. The results suggest a potential anti-inflammatory instead of the original hypothesized pro-inflammatory role of IL-6 signaling in the *mdx* mice.

## Background

A hallmark feature of Duchenne Muscular Dystrophy (DMD) is chronic inflammation, it occurs within the muscle of patients and in the mouse model of the disease (*mdx*). Several inflammatory factors (cytokines) increase in the muscle including IL-6, TNF-alpha, and IL-1 beta [1]. Additionally inflammatory cell infiltration is found in DMD and *mdx* muscle, the majority of these cells being macrophages and lymphocytes [2]. And yet, while the primary protein defect (dystrophin) causes the disease, it is now believed that the chronic inflammation in skeletal muscle hinders the repair or regenerative process that would occur if this inflammation was reduced [1,3,4]. Indeed, corticosteroids are a current standard therapy for boys with DMD, which may act by reducing inflammation. Yet corticosteroid effects are highly variable, last for a limited period of time, and have severe adverse side effects [5]. The exact mechanism by which corticosteroids benefit these patients is unknown and somewhat paradoxical; long term use causes muscle wasting in most clinical populations except DMD. If the beneficial effects (e.g. anti-inflammatory) of corticosteroid use could be realized without the side effects it could provide a treatment for those with DMD. As inflammation is seen as a primary contributor to the disease pathology, identifying the molecular mediators (e.g. cytokines, inflammatory cells, growth factors) of inflammation that are beneficial to muscle repair in DMD pathology is likely to lead to new treatments.

Several studies have examined the effect of blocking inflammatory cytokines or depleting inflammatory cells in *mdx* and have consistently yielded promising results. [2,4,6-10]. For example, in a model of T and B cell depletion the *scid* mouse was recently crossed with the *mdx* (*scid/mdx*) [11]. Scid/mdx mice showed significantly improved muscle pathology as measured by fibrosis, muscle necrosis, and TGF-Beta production. Yet, a report of immune system depletion (R*AG2/mdx* mouse) produced no improvement in muscle strength or function [12]. Paradoxically, reducing inflammation in healthy animals where muscle damage has occurred is detrimental to tissue repair [13] and satellite cell activation and differentiation [14]. Thus it seems a reduction, not absence of inflammation is ideal. In this regard, specific mediators and markers of the inflammatory process (e.g. IL-6) warrant examination in a dystrophic model.

Il-6 is a ubiquitously expressed cytokine that can have pro and anti-inflammatory effects depending on concentration and the local tissue milieu of immune cells and cytokines. IL-6 exerts its biological activities through interaction with specific receptors expressed on the surface of target cells. The receptor complex mediating the biological activities consists of two distinct membrane-bound glycoproteins (ligand binding gp80 and non-ligand binding component gp130). As numerous cells of the body express the gp130 transmembrane receptor, the activity of IL-6 is wide spread. However, the IL-6 receptor is required for gp130 activation. The IL-6 receptor (IL-6r) can be found on the cell surface or in soluble form. IL-6 binding to its soluble receptor and then interacting with the gp130 receptor is known as “trans” signaling and allows amplification of IL-6 signaling to cells not normally expressing the specific receptor. The soluble form of the receptor can also be shed from cells or be expressed by alternative splicing in cells where IL-6r is expressed. Thus numerous cell / tissue types can be affected. By blocking the IL-6r binding site, IL-6 cannot activate gp130, which effectively eliminates most IL-6 signaling throughout the body. A monoclonal antibody (mAb) for IL-6r has been successfully used to prevent and reverse Crohn’s disease, rheumatoid arthritis, and other inflammatory diseases by suppressing inflammatory events [15-17]. Indeed, our collaborators laboratory recently utilized IL-6 blockade through this same antibody to suppress the muscle protein degradation of cancer cachexia [18]. And while all of the mechanistic actions of IL-6 blockade are not yet clear, it is very likely to reduce systemic and local levels of inflammation, possibly affecting growth factor production (i.e. Insulin like growth factor 1 (IGF1)) and thus tissue growth and maintenance [19-23]. Indeed, transgenic mice overexpressing IL-6 suffer from severe muscle atrophy which can be ameliorated by blockade of IL-6 with IL-6r antibody [22]. Likewise, in the *mdx* mouse IL-6 muscle levels are dramatically elevated, while in age matched wild-type IL-6 is negligible; indeed, it’s barely detectable via western blot in wild-type mice [24]. Interestingly, muscle of wild-type mice, when injured, expresses IL-6 at a similar level to *mdx* mice, but control animal IL-6 levels quickly return to baseline. Thus, increased levels of IL-6 in muscle are a normal response to injury but resting levels are very low in healthy muscle and very high in *mdx* muscle. In humans, acute increases in IL-6 will decrease circulating IGF1 (muscle growth factor), while chronic high levels of circulating IL-6 are associated with a decreased skeletal muscle mass [25,26]. The effect on IGF1 is likely direct because IL-6 directly affects liver and muscle IGF1 [27,28]. Finally, primary muscle cell proliferation and differentiation (known to be critical for muscle growth) are modulated by IL-6 [27,29,30]. Thus again while some IL-6 is necessary for proper cell function, chronic high level exposure of IL-6 to muscle cells seems to be detrimental. Due to the known effect of IL-6r mono-clonal antibody (mAb) on inflammation, muscle atrophy, and the relationship of inflammation to muscle function in *mdx* mice as mentioned, it is an ideal candidate therapeutic agent to test in the *mdx* mouse.

In this study, we tested the efficacy of this agent in mitigating muscle disease in *mdx* mice. As IL-6 is likely to have systemic and local effects, we examined these through direct (Suppressor of cytokine signaling 3 (SOCS3), Serum amyloid a (SAA)) and indirect (TNF-alpha, IGF1) targets of IL-6 signaling in target tissues, liver and muscle. And as chronically elevated levels of IL-6 have most often been reported to be detrimental to skeletal muscle health, we hypothesized that IL-6r mAb treatment will reduce chronic muscle inflammation and necrosis and improve muscle function. Additionally, we hypothesize that these therapeutic effects will be related to the effect of IL-6 on IGF1 production.

## Methods

### Experimental protocol

Thirty-two male mice, 16 C57BL/10ScSn-Dm (*mdx*) and 16 C57BL/10SnJ (BL-10) mice purchased from Jackson Laboratories were used in this study. Mice arrived at our laboratory at approximately 4.5 weeks of age and were acclimatized to their new environment for 3 days. BL-10 and *mdx* mice were randomly assigned to either IL-6r mAb or iso-type matched control (Kh-5) injection groups. To induce tolerance as previously reported for these drugs, all mice were given an initial bolus injection (200 mg/kg in 200 μl sterile saline) of either IL-6r mAb or the Isotype-matched control (Kh-5) in order to avoid neutralizing antibody production [31]. Mice were then given subcutaneous injections of their corresponding treatment (IL-6r mAb or Kh-5), every third day, 5 mg/kg in 200 μl sterile saline solution for five weeks (8 mice/group and four groups). The experimental drugs, anti-IL-6r mAb (clone MR16–1, rat IgG1) or purified rat IgG as control (KH-5) were a kind gift of (Chugai-Roche Co. Ltd).

Animals were housed at the Veterans Affairs Animal Research Facility and all protocols were approved by The Children’s National Medical Center and V.A. IACUC, Washington, DC. Mice were checked daily for signs of distress and were maintained on a 12:12-h light–dark cycle in a low-stress environment (22°C, 50% humidity and low noise) and given food and water ad libitum. Data collection of performance measures were performed within three hours of the end of the active dark cycle (0700). After final data collection (24 hours after final subcutaneous injection), mice were asphyxiated with CO2 and muscles were immediately dissected, washed in PBS, blotted, weighed and frozen in isopentane cooled with liquid nitrogen. All samples were transferred to chilled tubes on dry ice and stored at -80c until analysis. Diaphragms were carefully extracted and placed directly in 10% buffered formalin for 24 hrs, transferred to 70% ethanol and shipped to HistoServe inc. (Germantown, MD) for paraffin embedding, cross-sectioning and H&E staining.

Rotarod (RR) Performance was used to assess neuromuscular coordination at the conclusion of the study. All mice underwent 3 days of acclimatization to the protocol before primary data collection. Mice were placed on the rod in the identical forward direction, and then the RR (Life Science Series 8) was started at 0 rpm and increased to 40 rpm at 0.4 rpm/s, after a 60 second acclimatization period at 4 rpm. The latency to fall from the rod was recorded in seconds with a maximum time of 180 seconds. This procedure was repeated for a total of six trials (2 trials per day with > 2 hours of rest time between trials) over three days, and the average of the trials was used in the data analysis.

Grip Strength Measurements was used to assess muscle strength, with measures taken after 5 weeks of treatment. All GSM measures were conducted by one trained investigator. For forelimb strength, holding the mice by the tail, the front feet were allowed to grip a grate, and then they were pulled from the grate, generating a force measured by the force transducer (Columbus Instruments). For hind limb strength, both front and back feet were allowed to grip the grate and mice were pulled across the grate. Five measurements were taken, five days consecutively, with the first day used as acclimatization and not included in final data analysis. The average of the measurements was used in the data analysis.

### Histology

To assess histopathologic differences of mice for skeletal muscle and diaphragm after treatment, standard measures of fiber diameter, central nucleation, muscle regeneration and inflammatory cell counts were made, similar to those suggested for *mdx* drug trials [32]. To quantify muscle histology, transverse frozen muscle sections were cut in a cryostat after partial embedding in OCT. Cryostat sections (12-μm thick) were cut at the mid-belly of the gastrocnemius of at least 2 sections with a minimum of 50 μm of separation between each section. Sections were stained with H&E, dehydrated in ethanol washes, rinsed in xylene, mounted with Permount, and dried overnight. Stained slides were observed under 400× magnification (Olympus BX41) with 2 experienced, blinded reviewers randomly selecting a total of 20 fields per mouse between at least 2 cut sections for analysis. Diaphragm analysis consisted of identical measures as skeletal muscle though fewer slides of optimally preserved tissue were available, which resulted in lower cell counts in the diaphragms. The Infinity Analyze software, 4.4 was used for all analysis. The total number of fibers was counted using the manual counting feature in the software for each of the 20 fields. All cells in the field of view were used in the analysis. Additionally, total myonuclei, centralized myonuclei, and regenerating fibers were counted in 15 fields. To distinguish inflammation, regenerating fibers, or fibers with central nucleation standardized criteria were used. Regenerating fibers were identified by small caliber, centralized large nuclei, and basophilic cytoplasm. Fiber diameters were calculated based on the minimal ‘Feret’s diameter’ [33]. Fibers not otherwise identified as regenerating or inflammatory that contained a centralized nuclei were counted as a centrally nucleated fiber. Finally, an inflammatory score was assigned to each field of view and averaged per slide (mouse), based on the number of observed mononuclear cells: (<5 inflammatory cells = 0, 5–10 inflammatory cells = 1, 11–15 = 2, and >15 =3). Data were expressed as absolute cell counts, μm (diameter), or inflammation score 0–3. As a global measure of the muscle damage and repair process (muscle remodeling), we combined the inflammation and regeneration data by first ranking the scores for these two variables using a rank-order scale for each mouse. This was based on scores for regeneration and inflammation for each individual animal. The rank-order scale allowed us to combine these data and utilize a non-parametric statistical analysis to compare between treatment groups and determine a percentage difference in rankings between treatment groups.

### ICAM-1 staining

Frozen *mdx* muscle (gastrocnemius) sections were cut in a cryostat as described for histology, (10-μm thick). Sections were collected on glass slides and air dried for 20 minutes, fixed in acetone/methanol (50/50) and rehydrated in PBS. Muscle sections were blocked in 10% HS, PBST for 1 hour; incubated with primary ICAM1 Ab (biotin labeled, Biolegend) 1:100 in blocking solution at 4 degrees overnight and washed three times (10 minutes) in PBST. Slides were then incubated with avidin (HRP, BioLegend) 1:500 in blocking solution for 60 minutes at room temperature. Tissue staining was completed with DAB substrate (Vector labs) according to the manufacturer’s protocol. DAB reactions were optimized and stopped at 3 minutes with cold tap water. Sections were dried briefly and covered with aqueous mounting medium and glass cover slip.

Sections were examined at 400× magnification (Olympus BX41). A trained blinded reviewer conducted analysis by selecting 20 random fields from the two muscle sections per mouse. Lumens were scored as positive or negative for staining, intra-tester reliability was determined for the one reviewer analyzing six random slides (Kappa agreement median = 0.93). Total number of lumens and total number of “positive” lumens were quantified. Data are reported as mean number of lumens and positive lumens per mouse. Normalization was conducted by dividing the number of positive lumens by the number of negative lumens per mouse and then averaging per treatment.

### Quantitative RT-PCR

The contralateral (from that used for histology) gastrocnemius and an ~30 mg section of liver from each mouse was used for RNA extraction and subsequent quantitative real-time PCR analysis. Tissue was homogenized with a Polytron homogenizer in 1 ml of Trizol (Invitrogen) and RNA was extracted per manufacturer’s instructions. Isolated RNA pellets were resuspended in 20 μl of DEPC treated water and RNA concentration, purity, and integrity were examined using 1 μl of RNA in the Nanodrop 1000 with a subsequent 800 ng of RNA run in a 1.1% RNase-free agarose gel stained with ethidium bromide. Pictures of gels were taken on an UV imager (BioSpectrum, UVP).

Total RNA (3 ug) was reverse transcribed to cDNA using oligo dT primer (0.05 ug/μl) and reagents (Superscript II) from Invitrogen, (CA) and according to manufacturers insturctions, cDNA was amplified in triplicate in SYBR® Green PCR Master Mix (Applied Biosystems, CA) except for TNF-alpha and IL-10 which were examined using TaqMan Gene Expression Assays per manufacturers instructions. The thermal cycling conditions of our ABI 7300 Real-time PCR system include 94°C for 5 minutes, followed by 40 cycles of amplification at 94°C for 30 seconds, followed by 60°C for 1 minute. Each SYBR Green reaction contained 20 ng of template cDNA with 75 nM primer concentration. PCR primers were designed using a Primer Express program v 1.01 (Applied Biosystems, CA). Primer sequences used for murine IGF1 were (forward) 5’ CGCTCTGCTTGCTCACCTTCAC 3’, (reverse) 5’ CACTCATCCACAATGCCTGTCTG 3’; SAA (forward) 5’ CTGCAGAAGTGATCAGC 3’, (reverse) 5’ ATTGTGTACCCTCTCCCC 3’; 36b4 (forward) 5’ GCAGACAACGTGGGCTCCAAGCAGAT 3’, (reverse) 5’ GGTCCTCCTTGGTGAACACGAAGCCC 3’; SOCS3 (forward) 5’ GATTTCGCTTCGGGACTAGCTC 3’, (reverse) 5’ TTGAGGCGCAGGCTGGTG 3’; GAPDH (forward) 5’ GGAGCCAAACGGGTCATCAT 3, (reverse) 5’ TCACGCCACATCTTTCCAGA 3’.

Standard controls were employed for all qRT-PCR amplifications. Glyceraldehyde-3-phosphate dehydrogenase (GAPDH) was used as internal control in the muscle samples while acidic ribosomal phosphoprotein (36b4) was used for liver samples. The primers were purchased from Applied Biosystem, CA. All primers were tested for non-specific amplicons and primer dimers by visualizing PCR products on 2% agarose gels prior to performing qRT-PCR, as well as by dissociation curve analysis following the RT-PCR assays. Additionally, no template controls were checked for each reaction by melting curve, agarose gel, and ΔRn. Primer, template, PCR efficiency was checked for each primer pair. The 2^ΔΔCT method was used to determine fold differences and a t-test was used (P < 0.05) to test statistical significance.

### Data handling and statistical analysis

Results from body weight are reported as means (SD). All other data are reported as means ± SE. Force production is reported in Newtons per gram of body mass (N/g) and RR is reported as latency to fall in seconds. Appropriate statistical assumptions (e.g. normality and variances) were examined before hypothesis testing. Data analysis comparing IL-6r mAb treated mice was done independently for *mdx* and BL-10 mice with two-tailed independent t-tests for analyzing central nucleation, inflammation score, fiber diameter, regenerating fibers, ICAM-1 lumen scores, and qRT-PCR. To examine muscle remodeling, quantification scores for inflammation and regeneration were converted to a rank-order scale, the data was pooled, and a Wilcoxon rank-sum test compared differences between treated and untreated mice. To maintain consistency of the intervention with respect to age of mice (the young age of mdx mice being critical to inflammation), a subset (n = 5 per group) of mice were tested at baseline to asses differences amongst treatment groups for RR and Grip strength, no differences were detected amongst treatment groups thus all analysis was conducted between groups on data collected at the conclusion of the study. The level of significance was set at P < 0.05 for all analyses. Statistical analyses were carried out using SPSS software (SPSS Inc., Chicago IL)., version 18.0.

## Results

**Figure 1:**
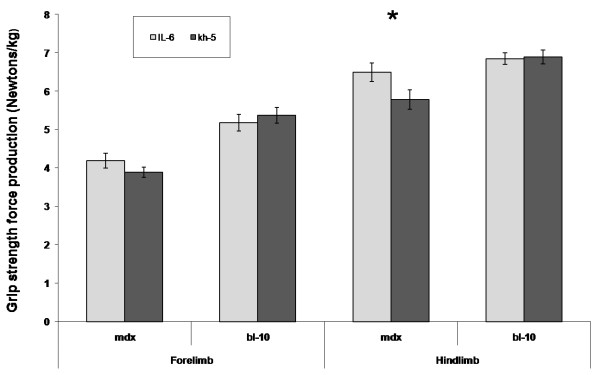
**Comparison of grip strength corrected for body weight after IL-6r mAb treatment.** Comparison of final grip strength measured in BL-10 and mdx mice after 4 weeks of IL-6 mAb or control Ab (Kh-5) treatment. T-test comparisons were made between treatment groups. Asterisk represents difference of (p = 0.055). Data are means and bars represent SEM.

All mice completed the IL-6r mAb trial. No adverse events occurred due to the multiple injections. At baseline, body weights between groups were similar and increased as expected over the 5-week trial. No differences were noted between IL-6r mAb and Kh-5 groups in total body or gastrocnemius dissection weight. Antibody treatment resulted in an 11 % greater strength increase (hindlimb) in IL-6r mAb treated *mdx* mice compared to Kh-5 treated *mdx* mice, though this did not reach statistical significance (6.5 ± 0.2 N/g versus 5.7 ± 0.3 N/g, P = 0.055). No difference was found in forelimb strength of *mdx* IL-6r mAb treated mice when compared to *mdx* Kh-5 treated (P = 0.28, Figure 1). In control mice (BL-10), no differences were noted between IL-6r mAb and Kh-5 treated mice (P = 0.53, 0.86 forelimb and hindlimb respectively).

### Rotarod

**Figure 2:**
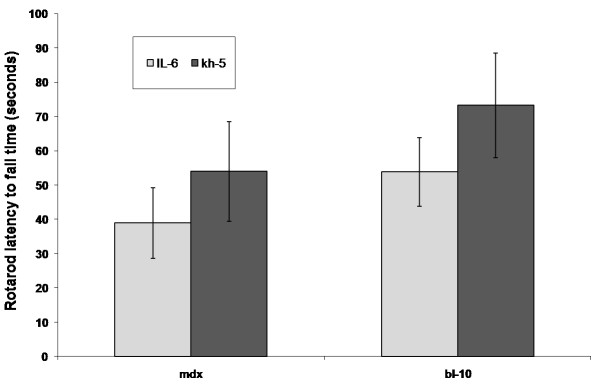
**Comparison of rotarod latency to fall time after IL-6r mAb treatment.** Comparison of BL-10 and mdx mice after 4 weeks of IL-6 mAb or control Ab (Kh-5) treatment. Data are means and bars represent SEM.

All mice completed RR measures with only one *mdx* mouse per group (IL-6 or Kh-5) attaining the 180 s maximum measure. In contrast, nine BL-10 mice achieved the 180 s maximum, five from the IL-6r mAb group and 4 in the Kh-5 group. No significant differences in RR performance (Figure 2) were noted after treatment between IL-6r mAb and Kh-5 treated *mdx* mice (P = 0.43) or BL-10 (P = 0.32).

### Histology

**Figure 3:**
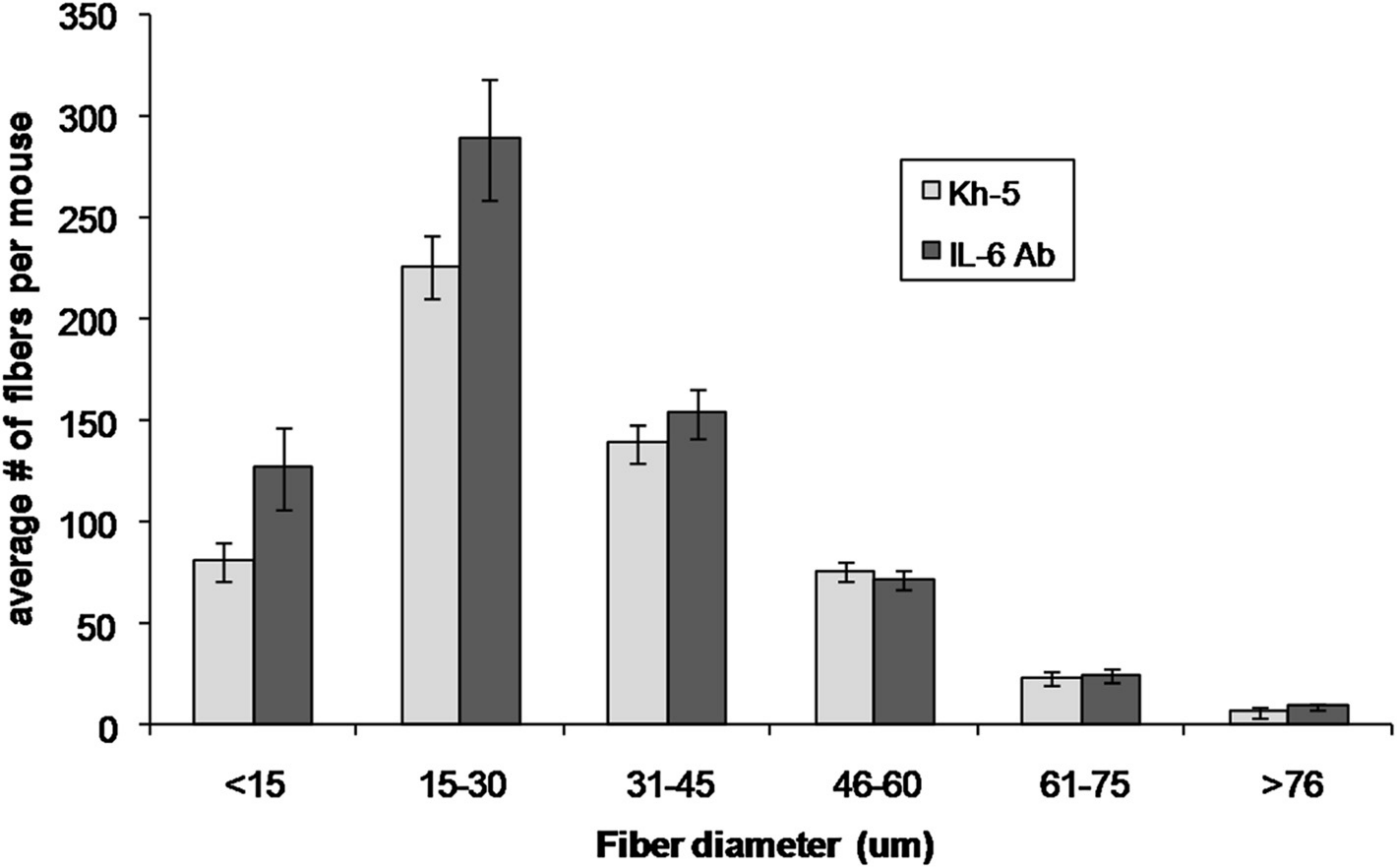
**Fiber diameter histogram of mdx mice after 4 weeks of IL-6r mAb or control (Kh-5) treatment.** Data are means and bars represent SEM.

Blinded reviewers analyzed the randomly selected microscopy fields of H&E stained sections resulting in approximately 840 and 780 muscle fibers analyzed respectively for each *mdx*-IL6r mAb and *mdx*-Kh5 mouse. For comparison, approximately 600 fibers from BL-10 mice were examined (Figure 1). There were no differences between BL-10 treatment groups. Measures of central nucleation (CN) were not significantly different between treated and un-treated *mdx* mice, though *mdx*-IL6r mAb treated mice had 8% (P = 0.27) fewer CN fibers in skeletal muscle gastrocnemius (Gas) (Table 1). Inflammation scores were nearly 50% higher in IL-6r mAb treatment group in skeletal muscle (Gas) as compared to *mdx* Kh-5 treated mice (P = 0.04). A histogram of muscle diameters (Figure 3) demonstrates little difference between IL-6r mAb treated and Kh-5 treated *mdx* mice, though IL-6r mAb treated mouse muscle diameters were slightly smaller (Table 1). IL-6r mAb treatment demonstrated no change in the number of regenerating fibers (P = 0.07) (Table 1). To understand the muscle damage and regeneration process from a global perspective, as IL-6 plays a complex role in the process of muscle degeneration/regeneration, we combine the inflammation and regeneration scores by first converting them to a rank-order scale, combining the two ranks, followed by a Wilcoxon rank-sum test. The results showed that the pooled variable of muscle remodeling increased 37% in IL-6r mAb *mdx* mice compared to Kh5 treated *mdx* (P = 0.02).

**Table 1:** Histological analysis of L-6r mAb treated and control (Kh-5) treated mdx and bl/10 mice

	**Regenerating fibers (n)**	**Diameter (um)**	**Central nucleated fibers (%) Gas**	**Central nucleated fibers (%) Dia**	**Inflamation score**
Kh5-mdx	5.6 ± 1.2	34.1 ± 1.0	72.8 ± 2.6	32.8 ± 1.9	14.9 ± 3.4
IL6rab-mdx	9.6 ± 1.7	31.7 ± 1.6	64.5 ± 6.9	29.3 ± 1.4	26.2 ± 3.1
kh5-bI/10	0.4 ± 0.1	38.2 ± 1.1	2.2 ± 0.5	1.8 ± 0.3	1.4 ± 1.2
IL6Ab-bl/10	0.2 ± 0.1	40.1 ± 0.8	1.5 + 0.5	0.9 ± 0.1	3.1 ± 1.1

Data are means and SEM for the gastrocnemius (Gas) unless indicated as diaphragm (Dia).

### ICAM1 staining

**Figure 4:**
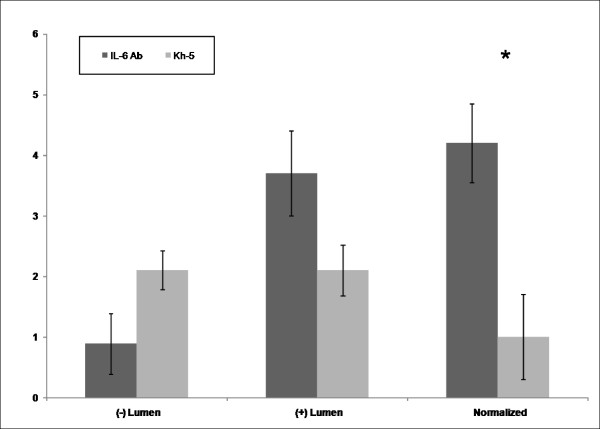
**Comparison of lumen counts for ICAM1 staining of gastrocnemius cross-sections.** Comparison of mdx mice after 4 weeks of IL-6r mAb or control Ab (Kh-5) treatment. * indicates P < 0.05. Data are means and bars represent SEM.

**Figure 5:**
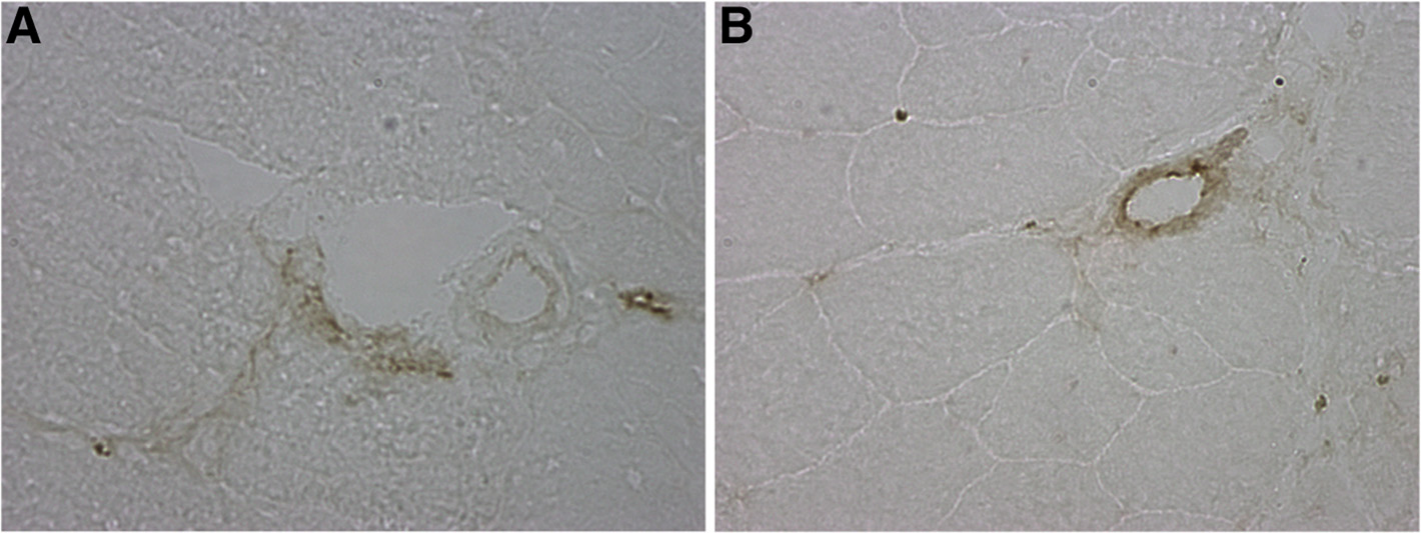
**Representative (+) and (−) stained lumens in mdx gastrocnemius muscle.A**) Negatively stained lumen **B**) positively stained lumen; (400× magnification). Scale bar: (A) 50 μm.

A total of 20 fields were randomly selected per mouse. A total of 38 lumens within the skeletal msucle were quantified for Kh-5 treated, and 41 lumens for IL-6r mAb treated *mdx* mice. Figure 4 demonstrates the differences between *mdx*-IL-6r mAb treated and *mdx*-Kh-5 treated. Figure 5 is representative of a positive versus negatively stained lumen for ICAM1. When normalized to the number of non-stained lumens, IL-6r mAb treated *mdx* had 4 positive lumens to Kh-5’s 1 (P = 0.04).

### Real-time PCR

**Figure 6:**
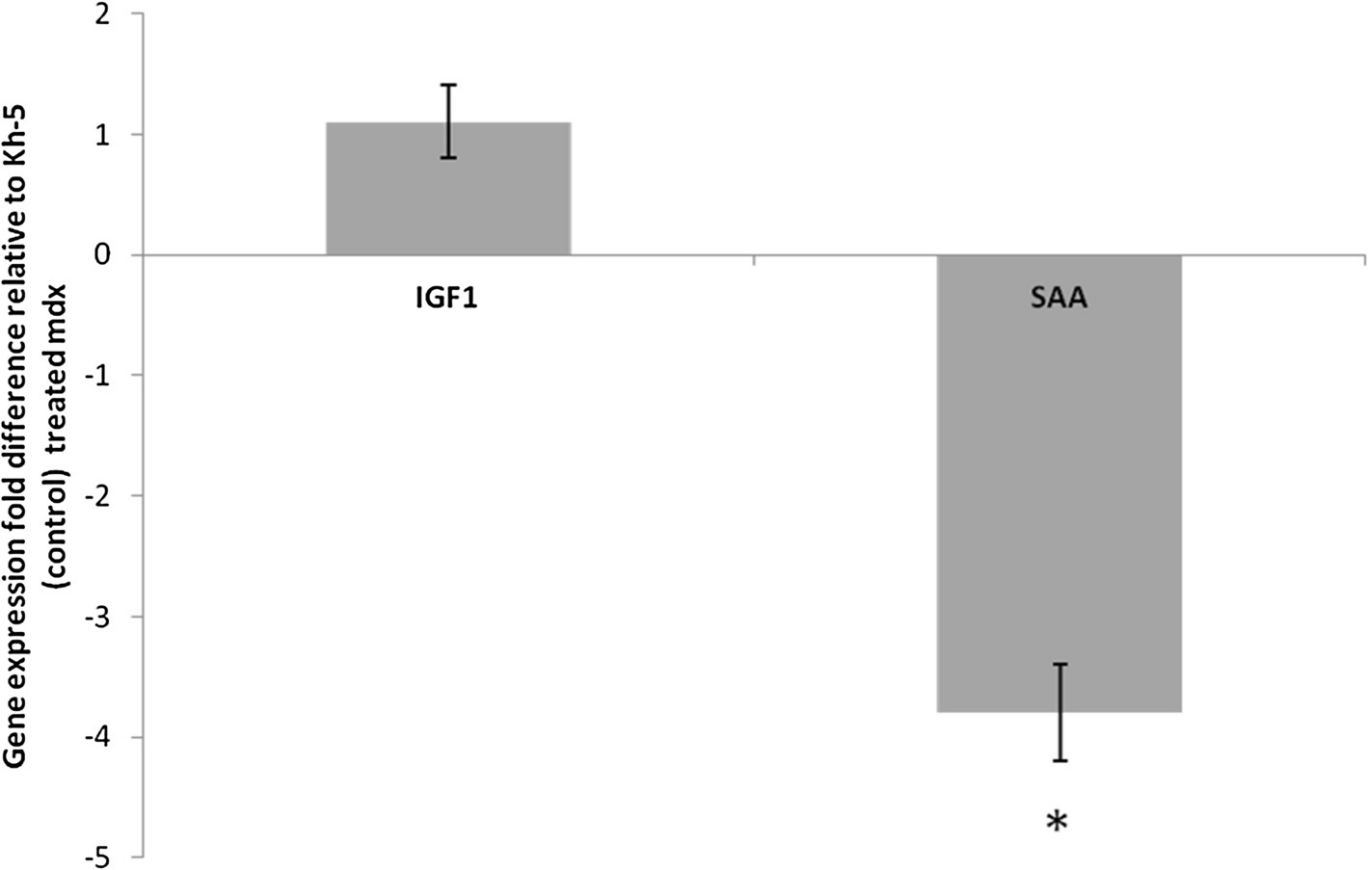
**Gene expression of mdx treated relative to control-Ab treated liver.** Insulin like growth factor 1 (IGF1) and serum amyloid A (SAA) were measured in liver samples of mdx mice treated with IL-6r mAb (blocks IL-6 signaling) or control Ab. Samples are normalized to GAPDH and the fold change from mdx control mice is presented. Independent t-tests examined statistical differences (* indicates p < 0.05). Data are means and bars represent SEM.

**Figure 7:**
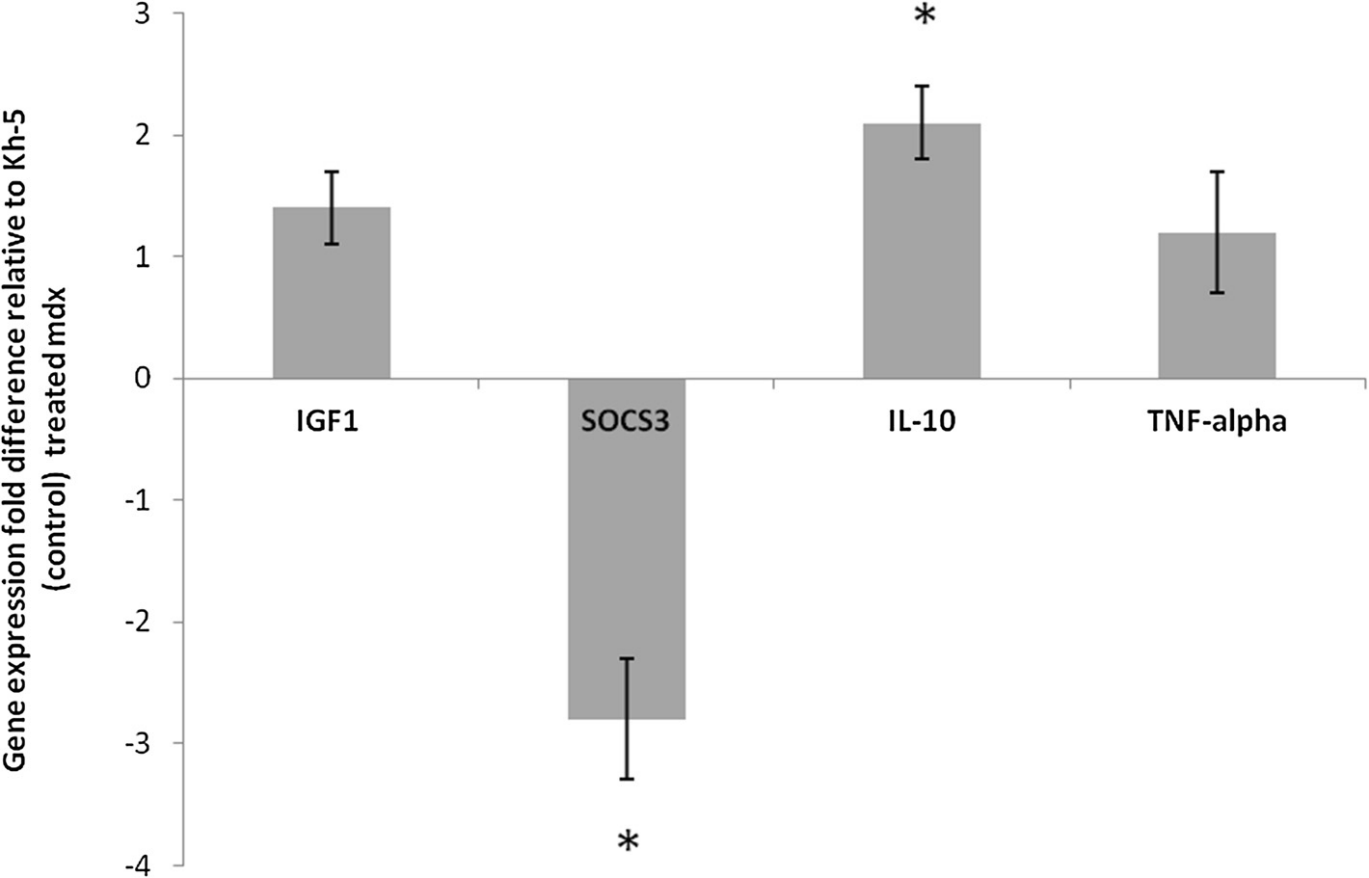
**Gene expression of mdx treated relative to control-Ab treated gastrocnemius muscle.** Insulin like growth factor (IGF1), Suppressor of cytokine signaling 3 (SOCS3), Tumor necrosis factor (TNF-alpha), and Interleukin 10 (IL-10) were measured in muscle samples of mdx mice treated with IL-6r mAb (blocks IL-6 signaling) or control Ab. Samples are normalized to GAPDH and the fold change from mdx control mice is presented. Independent t-tests examined statistical differences (* indicates p < 0.05). Data are means and bars represent SEM.

Under optimized conditions for all primer pairs, SAA mRNA (marker of systemic IL-6 action) levels in the liver were 3.8 ± 0.4fold lower (P < 0.01) in *mdx*-IL-6r mAb treated mice versus *mdx*-Kh-5 treated mice (Figure 6). Gastrocnemius muscle SOCS3 (marker of local IL-6 action, Figure 7) was 2.8 ± 0.5 fold lower (P = 0.05) and IL-10 was 2.1 ± 0.3 fold higher (P = 0.03) in *mdx*-IL-6r mAb treated mice versus *mdx*-Kh-5 treated mice (Figure 6). TNF-alpha of treated *mdx* was not different from untreated *mdx*, (1.2 ± 0.5 fold, P = 0.45). IGF1 mRNA levels were not significantly different between *mdx*-IL-6r mAb and *mdx*-Kh-5 treated mice in the liver, (1.1 ± 0.1 fold higher in *mdx*/IL-6r mAb, P = 0.78) or muscle (1.4 ± 0.3 fold higher in *mdx*/IL-6r mAb, P = 0.71, Figures 6 &7).

## Discussion

Here we report the first study to block IL-6 signaling in *mdx* mice. We hypothesized that overproduction of IL-6 by *mdx* muscle contributes to the pathologic inflammation of the disease and that by reducing IL-6 signaling we would decrease inflammation and improve muscle pathology. On the contrary, we report that five weeks of IL-6 blockade caused an increase in muscle inflammation, with no change in muscle regeneration. Also contrary to our expectations, we found no change in IGF1 gene expression by muscle or liver. And although there was a trend for improvements in one measure of grip strength after 5 weeks of IL-6r mAb treatment, no measures of muscle function reached statistical significance. The increase in inflammation is complex and will require a more specific inquiry into mechanisms and consequences than our current study provides. The modulation of inflammation we observed could be beneficial (e.g. up-regulation of IL-10) or detrimental.

Chronic high levels of circulating IL-6 is associated with muscle wasting in rodents and humans. Exogenous injections of IL-6 induces skeletal muscle protein breakdown in rats [34] and mice [35]. In addition, skeletal muscle growth in young rats is attenuated by chronic high levels of IL-6 [36]. In terms of disease states, overexpression of IL-6 can cause cancer cachexia [37]. In humans, exogenously administered IL-6 up to high physiologic circulating levels, causes a decrease in circulating IGF1 [26]. The decrease in IGF1 is likely due to decreased liver production and this is likely to attenuate muscle growth. Additionally, in cross-sectional studies of the elderly, a decrease in muscle (sarcopenia) correlates with high circulating IL-6 [25]. Yet, while chronic high levels of IL-6 appear detrimental to muscle, a complete absence of IL-6 hinders muscle repair. Knock-out models of IL-6 have shown that it is necessary for proper muscle hypertrophy, activation of satellite cells, and regulation of extracellular matrix remodeling [38,39]. Thus the IL-6/muscle interaction is complex but chronic high levels of IL-6, as occurs in the *mdx* mouse, is likely detrimental to muscle [24].

Therefore, as chronic inflammation is associated with muscle loss and reducing inflammation in *mdx* appears to be beneficial, we used a compound that blocks a mediator of systemic (acute phase response, APR) and muscle inflammation, IL-6. The IL-6r mAb has been used successfully to treat several models of inflammatory diseases [15-17]. In each trial, circulating IL-6 protein levels are not affected but IL-6 signaling is blocked as verified by common liver markers of APR such as serum amyloid A (SAA) expression. Therefore, we verified effectiveness in our model by examining the immediate downstream effect of IL-6 signaling systemically (SAA in liver) and locally (SOCS3 in muscle). Indeed, IL-6r mAb had an effect both systemically and locally to reduce IL-6 signaling in *mdx* mice

Contrary to our primary hypothesis, after 5 weeks of IL-6r mAb treatment in *mdx* mice, inflammation increased. Because IL-6 can increase or exacerbate the inflammatory process and because *mdx* mice have extremely high levels of IL-6 expression in skeletal muscle along with inflammation, we hypothesized that by decreasing IL-6 signaling, inflammation would also decrease. Analysis of H&E stained muscle samples found a nearly 50% increase in inflammation. Yet, H&E stained samples only estimate inflammation. To examine a more direct marker of tissue inflammation we quantified ICAM-1 as it will be expressed by the vascular endothelium where leukocytes will bind ICAM-1 on the endothelial surface and transmigrate across the vascular endothelial and into tissues during the inflammatory process [40,41]. ICAM-1 staining confirmed that an increase in inflammation occurred in our IL-6r mAb treated *mdx* mice. As this was contrary to our original hypothesis and due to the ability of IL-6 to modulate pro-inflammatory cytokines (TNF-alpha) while not affecting pro-growth cytokines (TGF-Beta), we sought to examine markers that are consistently shown to be pro-inflammatory or pro-growth in skeletal muscle. TNF-alpha muscle gene expression was not different between treated and untreated mice while IL-10 muscle gene expression increased. Thus it is possible that while overall inflammation increased it could be pro-growth and support increased regeneration (i.e. # of regenerating fibers), [42]. Indeed, IL-10 has been shown to be beneficial in *mdx* mice [43] and thus may be increasing regeneration, while no change in TNF-alpha is suggestive that overall pro-inflammatory signals were not affected by our treatment.

Our findings in inflammation are not without precedent, in *mdx* mice, other treatments that should decrease inflammation have been shown to cause an increase of inflammation [7,8]. In our current studies, this could have been caused by an increase in cytokines that directly bind to gp130 (e.g. IL-11, IL-27). Furthermore, IL-6 itself can be pro- and anti-inflammatory [44]. It is possible that IL-6 was increased in response to the high level of inflammation in *mdx* muscle and that our treatment was blocking the anti-inflammatory effects of IL-6. Also as mentioned, the increased level of inflammation may be pro-growth (e.g. 2 C macrophages) as evidenced by an increase in IL-10. The exact cause or implication of the increased inflammation is not clear. Yet, it is seemingly associated with an increase in regenerating fibers. And while our measures of regenerating fibers (H&E) were not statistically different, it was nearly double when comparing treated to untreated *mdx*. To examine this process from a global perspective that combines the overall damage/inflammation/repair process, we pooled inflammation and regeneration data as an indicator of muscle remodeling, and found a significant increase occurred due to IL-6 blockade treatment. While the consequences of this increase in remodeling are unknown, it is likely to have a long term effect as it is an indication of the overall tissue damage and repair process including critical aspects of muscle satellite cell activation. Thus while long-term effects are not known, IL-6 blockade did cause an increase in inflammation.

A marker of general muscle growth/repair, IGF1, can be directly affected by IL-6 levels [26]. As our mAb treatment should affect cell signaling and thus gene expression, we used IGF1 mRNA as an indicator of downstream consequence of blocking IL-6 signaling and an indicator of muscle growth. IL-6r mAb did not affect IGF1 gene expression in muscle or liver of *mdx* treated mice. And though IGF1 did not change, the increased remodeling is suggestive of additional growth factor release. Indeed, IL-10 did increase due to treatment and it is possible that other growth factors may have increased and/or macrophage subtype conversion to a pro-growth phenotype (M2c) could have occurred which leads to satellite cell or fibroblast proliferation [45]. Yet, in the case of fibroblast proliferation in *mdx*, it is likely to have detrimental consequences of increased collagen production and fibrosis [46]. The current treatment of 5 weeks is unlikely to produce any definitive results in terms of fibrosis; a longer study is needed. IGF1 gene expression was not affected locally or systemically, contrary to our hypothesis. This suggests that IL-6 was not hindering this growth pathway.

After 5 weeks of IL-6 signaling blockade in *mdx* mice there was no change in measures of muscle function. And while five weeks is a short period to improve muscle function, our a-priori calculation of statistical power should have allowed us to detect a 10% increase in some measures of muscle function. A post-hoc analysis of our data revealed a greater variance in our grip strength data than was used in our a-priori power calculation, causing a decrease in statistical power. In this regard, though the increase was not statistically significant, we note a trend for hind-limb grip strength to increase (11%). The increase in muscle strength, in our data, could be due to an actual increase in muscle force production or could be due to a reduction in body weight and while our *mdx* did tend to gain weight at a faster rate than BL-10 mice (which is typical of *mdx*) if body weight is increasing proportionally to the increase in strength, then functionally or in terms of ambulation, it would be of little consequence. Also noteworthy of the muscle strength testing is that testing could in theory cause muscle damage but we are aware of no reports of this affecting these measures in *mdx* mice. Furthermore, it was recently reported that although susceptible to injury, *mdx* mice respond to muscle damage by a repair process that is at least as efficient as BL-10 mice [47]. Our other measure of muscle function, neuromuscular coordination, could also have been improved by the reduced inflammation alone, though we found no change [48,49]. A more precise measure of muscle force production would have allowed us to detect smaller changes in muscle function and though this was attempted on a small number of soleus muscle samples (ex-vivo) technical difficulties in data collection prohibited analysis of. As improved muscle function could be caused by increases in size or number of myofibers, a reduction in muscle necrosis, or improved muscle repair we note that dissected muscle weights normalized for body weight were not different between groups of treated mice and while muscle fiber counts were greater in *mdx* treated mice in randomly selected microscopic fields, the fiber diameter was less and could account for the greater number of fibers counted. Thus as remodeling was increased it did not affect standard measures of muscle function.

The strength of the current study is the well characterized therapeutic agent and model of disease (*mdx*). The therapeutic agent has been used to treat several inflammatory diseases and blocks nearly all IL-6 signaling throughout the body and did so in our current investigation [15-17]. The early life of the *mdx* mouse (4–12 weeks) is considered the point of greatest inflammation and muscle necrosis and is thus an appropriate target age for studies such as ours [50]. A limitation of our study is having only one time point (5 weeks) of pathologic analysis. Thus, short-term effects beyond inflammation are difficult to interpret in terms of consequences such as fibrosis, longevity, or long term muscle function. Future studies should pursue the inflammation by more direct measures, i.e. cell sorting on fresh tissues, to more precisely quantify inflammation. Furthermore, a long term study could more directly address the effects of modified inflammation, fibrosis, and muscle repair.

## Conclusion

The current study showed that while IL-6 signaling is believed to contribute to the chronic inflammation and muscle wasting in DMD, reducing IL-6 signaling using neutralizing antibodies did not reduce the inflammation in *mdx* mice. Instead, the inflammation was increased by the treatment. Considering the muscle strength and pathology were either slightly improved or no change, the increase in inflammation is likely directly in response to the reduction of IL-6 signaling, which implies an anti-inflammatory role of IL-6 signaling in the *mdx* mice. In conclusion, while IL-6 is affecting the muscle inflammation and remodeling process in *mdx*, short term blockade does not produce statistically significant improvements in disease pathology or muscle function.

## Abbreviations

IL-6: Interleukin 6; IL-6r mAb: Monoclonal antibody against the IL-6 receptor; mdx: Dystrophin deficient mouse; TGF-B: Transforming growth factor – beta; IGF1: Insulin-like growth factor 1; RR: Rotarod; Gas: Gastrocnemius; 36b4: Acidic phosphoprotein; SAA: Serum Amyloid A; SOCS3: Suppressor of cytokine signaling 3.

## Competing interests

The authors report no competing interest.

## Authors’ contributions

MK contributed to all aspects of study design, data collection, interpretation, and manuscript preparation, KN contributed to study design, direction of data collection, and manuscript preparation, EP assisted with data collection and manuscript preparation, A assisted collected and analyzed all functional measures, SL assisted with data collection and manuscript preparation, BG assisted with data collection and manuscript preparation, YC contributed to all aspects of study design, oversaw data collection, and contributed to manuscript preparation. All authors read and approved the final manuscript.

## Grant support

NIH/NICHD R01HD048051 (Kostek and Chen), NIH/NIAMS R01 AR052027 (Chen), NIH/NICHD U54 HD053177 (Chen), NIH/NICHD R24HD050846 (Chen).

## Pre-publication history

The pre-publication history for this paper can be accessed here:

http://www.biomedcentral.com/1471-2474/13/106/prepub
